# Machine learning models to predict disease progression among veterans with hepatitis C virus

**DOI:** 10.1371/journal.pone.0208141

**Published:** 2019-01-04

**Authors:** Monica A. Konerman, Lauren A. Beste, Tony Van, Boang Liu, Xuefei Zhang, Ji Zhu, Sameer D. Saini, Grace L. Su, Brahmajee K. Nallamothu, George N. Ioannou, Akbar K. Waljee

**Affiliations:** 1 Michigan Medicine, Department of Internal Medicine, Division of Gastroenterology and Hepatology, Ann Arbor, Michigan, United States of America; 2 Department of Medicine, Veterans Affairs Puget Sound Healthcare System and University of Washington, Seattle, WA, United States of America; 3 VA Ann Arbor Health Services Research and Development Center of Clinical Management Research, Ann Arbor, Michigan, United States of America; 4 Department of Statistics, University of Michigan, Ann Arbor, MI, United States of America; 5 Michigan Medicine, Department of Internal Medicine, Division of Cardiology, Ann Arbor, Michigan, United States of America; 6 Michigan Integrated Center for Health Analytics and Medical Prediction (MiCHAMP), Ann Arbor, MI, United States of America; 7 Division of Gastroenterology, Department of Medicine, University of Washington, Seattle, WA, United States of America; 8 Division of Gastroenterology, Department of Medicine, Veterans Affairs Puget Sound Healthcare System, Seattle, WA, United States of America; Universita degli Studi di Pisa, ITALY

## Abstract

**Background:**

Machine learning (ML) algorithms provide effective ways to build prediction models using longitudinal information given their capacity to incorporate numerous predictor variables without compromising the accuracy of the risk prediction. Clinical risk prediction models in chronic hepatitis C virus (CHC) can be challenging due to non-linear nature of disease progression. We developed and compared two ML algorithms to predict cirrhosis development in a large CHC-infected cohort using longitudinal data.

**Methods and findings:**

We used national Veterans Health Administration (VHA) data to identify CHC patients in care between 2000–2016. The primary outcome was cirrhosis development ascertained by two consecutive aspartate aminotransferase (AST)-to-platelet ratio indexes (APRIs) > 2 after time zero given the infrequency of liver biopsy in clinical practice and that APRI is a validated non-invasive biomarker of fibrosis in CHC. We excluded those with initial APRI > 2 or pre-existing diagnosis of cirrhosis, hepatocellular carcinoma or hepatic decompensation. Enrollment was defined as the date of the first APRI. Time zero was defined as 2 years after enrollment. Cross-sectional (CS) models used predictors at or closest before time zero as a comparison. Longitudinal models used CS predictors plus longitudinal summary variables (maximum, minimum, maximum of slope, minimum of slope and total variation) between enrollment and time zero. Covariates included demographics, labs, and body mass index. Model performance was evaluated using concordance and area under the receiver operating curve (AuROC). A total of 72,683 individuals with CHC were analyzed with the cohort having a mean age of 52.8, 96.8% male and 53% white. There are 11,616 individuals (16%) who met the primary outcome over a mean follow-up of 7 years. We found superior predictive performance for the longitudinal Cox model compared to the CS Cox model (concordance 0.764 vs 0.746), and for the longitudinal boosted-survival-tree model compared to the linear Cox model (concordance 0.774 vs 0.764). The accuracy of the longitudinal models at 1,3,5 years after time zero also showed superior performance compared to the CS model, based on AuROC.

**Conclusions:**

Boosted-survival-tree based models using longitudinal information are statistically superior to cross-sectional or linear models for predicting development of cirrhosis in CHC, though all four models were highly accurate. Similar statistical methods could be applied to predict outcomes in other non-linear chronic disease states.

## Introduction

Progression to cirrhosis and its associated complications is of primary concern for patients with chronic liver disease. Rates of progression to cirrhosis can vary dramatically across individuals.[1, 2] Lack of accurate risk stratification can result in slow progressors undergoing excess monitoring and rapid progressors undergoing insufficient monitoring and treatment. Availability of highly accurate risk prediction models would facilitate proactive identification of individuals in need of more intensive monitoring and treatment. Risk prediction models would be particularly useful for application among individuals with chronic hepatitis C (CHC), a leading cause of cirrhosis worldwide. Despite availability of effective antiviral treatment for CHC, disease elimination remains quite challenging due to limited access to specialty care and the steep cost of antiviral medications. Furthermore, rates of new hepatitis C virus (HCV) infection are on the rise due to the ongoing epidemic of injection drug use. CHC remains undiagnosed in many patients, while others with known HCV lack of access to care or face treatment barriers such as cost concerns or substance use disorders. The ability to predict risk of CHC progression would help to identify patients at risk for adverse outcomes and could potentially help target resources to those at highest risk.[3,4]

Classic prediction models rely primarily on cross-sectional (CS) data and have limited accuracy to assess risk of disease progression in non-linear disease states.[5] Machine Learning (ML) algorithms provide effective methods to incorporate longitudinal data for risk prediction without over-fitting the model. Our group has previously shown that risk predictions for adverse outcomes among patients with CHC are more accurate when longitudinal data is incorporated and when used for pure classification problems with discrete and pre-specified time intervals.[5–7] We previously evaluated risk of fibrosis progression and clinical outcomes in CHC used longitudinal data from the Hepatitis C Antiviral Therapy to Prevent Cirrhosis (HALT-C) trial, which exclusively included patients with advanced liver disease who had failed prior antiviral treatment. We subsequently validated these models in a small cohort of individualswith minimal or no fibrosis and without prior treatment exposure. It remains unknown whether predictive modeling can accurately predict CHC progression in a large or heterogeneous sample outside of clinical trial settings. Further, in our prior studies, there is a possibility of not obtaining the best prediction accuracy if we treat the cirrhosis outcome as a dichotomous outcome at a specified interval of time. Thus, in this analysis we sought to address these questions by applying a time-to-event analysis to provide a more accurate approximation of the event over a continuous time period.

The Veterans Health Administration (VHA) cares for one of the largest cohorts of CHC patients in the US. VHA maintains a universal, longitudinal record of clinical services for all patients in its care. The aim of this study was to develop and subsequently validate models for risk of progression to cirrhosis among Veterans with CHC. In order to construct an accurate time-to-event risk prediction model, we compared performance of models using CS versus longitudinal data, and (regularized) linear Cox proportional hazards models versus boosted-survival-tree based proportional hazards models.

## Patients and methods

### Data source

The VHA is the largest integrated healthcare system in the US currently serving more than 8.9 million Veterans at 168 VA Medical Centers and 1,053 outpatient clinics throughout the country[8]. The VHA uses a single, nationwide, comprehensive electronic healthcare information network (known as the Veterans Information Systems and Technology Architecture or VistA), which consists of nearly 180 applications of clinical, financial, administrative and infrastructure needs integrated into a single, common database of all Veterans’ health information. We obtained electronic data on all patients who initiated antiviral treatment in the VA system using the VA Corporate Data Warehouse (CDW), a national, continually updated repository of data from VistA developed specifically to facilitate research[9]. Data extracted included all patient pharmacy prescriptions, demographics, inpatient and outpatient visits, problem lists, procedures, vital signs, diagnostic tests, and laboratory tests.

### Study population and data collection

**Fig 1:**
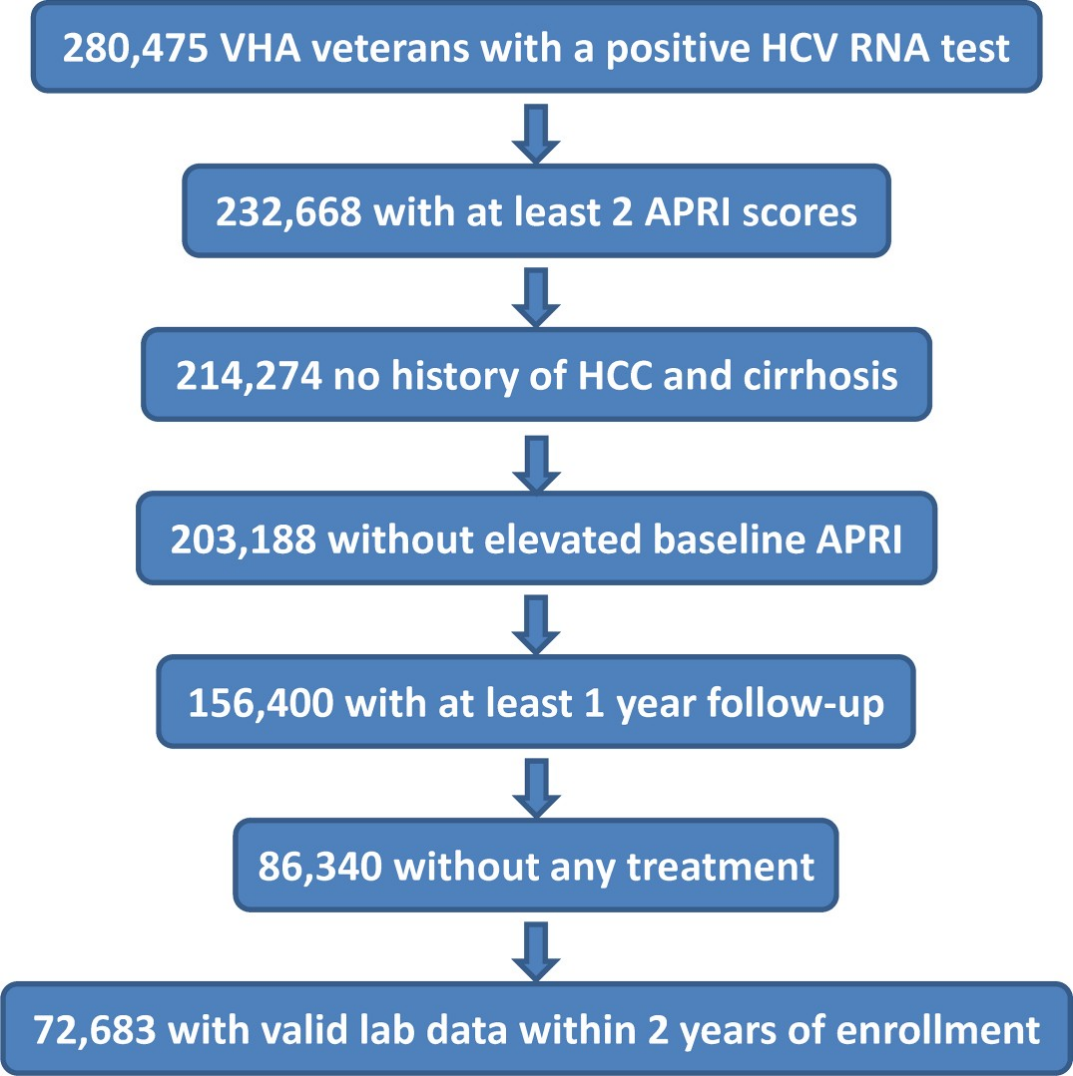
Study population.

Data were obtained from the VHA Corporate Data Warehouse spanning the period from January 1, 2000 through December 31, 2016 for all domains. Among 280,475 patients with at least one positive HCV ribonucleic acid (RNA) test result during this period, 232,668 had at least 2 APRI scores. A total of 214,274 had no history of HCC or cirrhosis prior to their first recorded APRI date, or within 1 year of their first APRI date. Among these individuals, 203,188 had initial first two APRI scores <2. Then, 156,400 individuals had a minimum of 1 year follow-up, and 86,340 patients received no HCV antiviral treatment. Finally, to leverage longitudinal information within a two year period, we further restricted to a subset of 72,683 patients that had valid lab measurements within 2 years of enrollment [**Fig 1**]. For the purpose of this study, we elected to design and test our prediction models only on patients without antiviral treatment given that antiviral treatment, particularly the older regimens, can impact laboratory values and thus may alter the performance of our models. In addition, patients with CHC without antiviral treatment provide the most representative population to evaluate the risk of fibrosis progression as this trajectory can be significantly altered among those who achieve sustained virologic response (SVR).

#### Outcome definition

Given the infrequent use of liver biopsy for fibrosis staging in clinical practice, progression to cirrhosis was defined using non-invasive biomarkers, specifically the aspartate aminotransferase (AST) to platelet ratio index (APRI). APRI has been shown to accurately assess fibrosis staging among patients with CHC, particularly with respect to detecting advanced fibrosis and cirrhosis.[10] APRI is defined by the formula APRI = 100 * (AST (U/L) / 40) / Platelet (1000/uL). The primary outcome of progression to cirrhosis was defined as the first occurrence of two consecutive APRIs > 2. The first APRI event within the consecutive pair was used as the index date for the outcome and serves as a surrogate for cirrhosis onset. Patient follow-up began on the date of the first recorded APRI, and ended on the first occurrence of any of the following events: (1) last recorded APRI, (2) the first APRI beginning a period of > 3 years of no APRI observations, signifying insufficient follow-up or drop-out, or (3) cirrhosis onset. Event types (1) and (2) are censored or non-events, and (3) is the outcome event.

#### Predictor variables

Predictor variables were selected based on results of our prior work and biologic plausibility. Given differences in lab reference ranges across different care sites, some labs were converted to a ratio of the lab value divided by the upper limit of normal (ULN) for the analysis. Laboratory tests included AST ratio, alanine aminotransferase (ALT) ratio, alkaline phosphatase (ALK) ratio, AST/ALT, APRI, albumin (ALB), total bilirubin (BIL), creatinine (CRE), blood urea nitrogen (BUN), glucose (GLU), hemoglobin (HEM), platelet count (PLT), white blood cell count (WBC), sodium (NA), potassium (K), chloride (CL), total protein (TOTP), and body mass index (BMI). All laboratory test variables are of longitudinal format. Demographic variables include patient age at enrollment, gender, and race.

**Fig 2:**
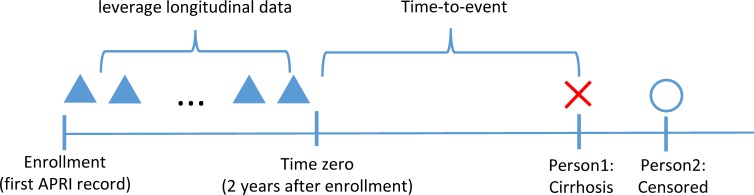
Model using longitudinal data.

We defined enrollment as entrance into the cohort at the date of the first APRI. Time zero was defined as 2 years after enrollment, to leverage longitudinal information between enrollment and time zero. We developed two categories of models: CS models and longitudinal models. CS models use predictors at or closest prior to time-zero, and longitudinal models use CS model predictors plus additional longitudinal variables [**Fig 2**].

To capture longitudinal information, we created five summary variables of longitudinal values between enrollment and time-zero: maximum, minimum, maximum of slope, minimum of slope, and total variation. The slope was defined as difference between two consecutive observed values divided by time difference, and total variation was defined as the average of absolute value of slopes. The average, average of slopes, and average of accelerations of laboratory test variables were tested but did not provide further improvement to prediction results. To avoid multicollinearity among predictors, these longitudinal summary variables were removed when fitting the model. We filled the missing values for the CS and longitudinal predictors in the training and testing data separately by median of available values.

#### Statistical analysis and model development

We choose to leverage the time-to-event nature of the data and utilize the survival analysis for predicting the onset of cirrhosis. Survival analysis allows us to predict the risk of developing cirrhosis within any continuous time window and therefore is more flexible than pure classification within a fixed discrete time interval. The time-to-event data consists of the triples {(x_i_,t_i_,δ_i_)}_i_, where x_i_ records the covariates that may affect the risk of the ith patient, and t_i_ records the time to the outcome δ_i_ (δ_i_ = 1 if the patient developed cirrhosis and δ_i_ =0 if the patient was right-censored). We used both a regularized linear Cox proportional hazards model and a boosted-survival-tree based proportional hazards model. [11,12] The reason we used the regularized t linear Cox model (rather than the standard Cox model) is that there are many predictor variables in our setting and many of them are highly correlated; regularization helps control the complexity and stability of the model and in general improves the model prediction accuracy and model interpretability. We also constructed tree based methods for model construction given that these models can handle mixturs of continuous and categorical predictor variables, allow non-linear effects of predictors and automatically perform variable selection. In the setting of survival analysis, survival-tree based ensemble methods such as boosted-survival-tree based proportional hazards model and random survival forests are widely used. [13,14] We did not use the random survival forests here as its computational cost is much higher than the boosted-survival-tree model given the size of the dataset. For each type of survival model, we fit a CS model using covariates at time zero and a longitudinal model with additional longitudinal predictors. In total four models were developed: CS Cox model, longitudinal Cox model, CS boosting model, and longitudinal boosting model. The negative and positive predictive values (NPV and PPV) of these models were reported to assess overall model performance.

#### Linear cox proportional hazards model

Survival models could be characterized by a hazard function, which is the probability that an outcome would happen at an instant given the patient has survived to that time. In Cox proportional hazards model, the hazard function takes the form h(t,x) = h_0_(t)exp{x^T^β}, where h_0_(t) is a baseline hazard rate changing with time t and exp{x^T^β} describes how covariates at time-zero, x, affect the hazard.[11] Cox model assumes that the hazard rate changes with unit increase in each covariate through a multiplicative effect. Given the hazard rate, we could obtain the survival function with respect to time, defined as the probability that a patient would develop the outcome after a specific time. Parameters in the Cox model are usually estimated through maximizing partial likelihood.

#### Variable selection for Cox models by elastic net

We performed variable selection for the CS and longitudinal Cox models using the elastic net approach.[15–17] Elastic net is a regularization and variable selection method which augments the original likelihood by a penalty which is a linear combination of the lasso and ridge penalties.[18,19] This method has the property of enforcing sparsity in the parameter estimation and thus allows variable selection similar as the lasso approach, while it can also accommodate a grouping effect where strongly correlated variables tend to be preserved or dropped from the model together. The grouping effect is very helpful in our models since the first-order predictors such as last value, maximum and minimum tend to be correlated. We did un-penalized Cox model analysis using the survival R package and performed Cox model with elastic net approach using the glmnet R package.[17,20]

#### Boosted-survival-tree based proportional hazards model

Boosted-survival-tree based proportional hazards model assumes covariates affect hazard rate through a non-parametric function in the exponential, i.e., h(t,x) = h_0_(t)exp{F(x)}.[13] The non-parametric nature of boosting allows the model to capture non-linear relationships and interactions and is more flexible. The target function F(x) is estimated by adapting the gradient boosting machine, where we search for the function that maximizes the partial likelihood in the function space rather than the parameter space.[21] At each iteration, F(x) is updated on the direction of its negative gradient, and the gradient function is usually fitted through a regression tree based on a random subsample of the training dataset. Such updates were performed over many iterations, and the final estimated function is chosen at the iteration when optimal out-of-bag prediction performance is achieved. We built boosting survival models using the gbm R package. We set the number of iterations as 2000 and restricted the interaction depth of trees as 2 so we could model up to two-way interactions among predictors.

#### Model performance: Concordance index and AuROC

We evaluated the overall performance of linear Cox models and boosting models using the concordance index. The concordance index, or C-index, is a global measure of discriminative power of a survival model. It is defined as the fraction of pairs of patients that the patient who has a longer survival time is also predicted with lower risk score by the model. The range of concordance is between zero and one, with a larger value indicating better performance (and 0.5 indicating discrimination by chance).

We also used area under the receiver operating characteristic curve (AuROC) to evaluate the performance at specific prediction windows, e.g., 1 year, 3 years and 5 years. Area under the ROC curve measures the probability that the classifier will assign a higher score to a randomly chosen positive outcome object than a randomly chosen negative one. To obtain 1 year AuROC, we predicted the risk at time 1 year and compared with patients’ true outcome at 1 year. Patients censored before 1 year were removed for such evaluation since their true outcomes at 1 year were not available. The 3 years AuROC and 5 years AuROC were calculated in a similar manner. Further, we selected the best cut-off point that is closest to the perfect point of both sensitivity and specificity equal one on the ROC plot.

#### Training and testing cohort

Training and testing sets were obtained by randomly splitting the data into 70% and 30% subsets. Such random splitting was performed 30 times. For each split we fit the linear Cox models and the boosting models on the training dataset and evaluated model performance on the testing dataset. The concordance index, 1 year AuROC, 3 years AuROC and 5 years AuROC were averaged over 30 replications. The 95% confidence intervals for all performance measures were derived from the 30 splits. The cut-off point selection and misclassification table were reported based on the split whose longitudinal boosting model has the closest concordance to the average of 30 splits.

#### Boosting variable importance

We evaluated the relative importance of each predictor in CS boosting model and longitudinal boosting model. The relative importance of each predictor is evaluated by aggregating the reduction of error in predicting the gradient at the nodes where the variable is used for splitting, across all trees generated by boosting. Variables that contribute more to error reduction have higher relative importance. The variable importance plots were plotted based on fitting boosting models on the entire cohort.

#### Partial dependence plots for boosting models

We constructed partial dependence plots for the CS and longitudinal boosting models to illustrate how individual predictors affect the log hazard, a quantity measuring patients’ risk of progression to cirrhosis. For each predictor variable, we used the boosting model fitted on the entire dataset to predict the log hazard for each patient with the predictor set as a given value and averaged over all patients to obtain a mean log hazard. This procedure was repeated for each present value of the predictor, or for its 50 quantiles if more than 50 values exist.

## Results

### Baseline characteristics and incidence of outcomes

The cohort consisted primarily of middle aged (mean 52.8 years) men (96.8%). The sample was primarily White (52.9%) or African American (40.7%). The mean duration of follow-up was 7 years. Baseline APRI was 0.62 with 16% of patients meeting the primary outcome over duration of follow-up [**Table 1**]. Summary statistics for baseline lab measurements uses the closest value to the patient enrollment date, but no more than 2 years after. Note that not every patient had an available lab measurement for every lab within two years of enrollment.

**Table 1:** Baseline characteristics.

Variable (N not-missing (% not-missing)) Cohort N = 72,683	Summary statistics
Primary outcome event (100%)	11,616 (16%)
Followup years (mean (sd)) (100%)	7.00 (4.01)
Age at enrollment (mean (sd)) (100%)	52.84 (8.74)
Male (%) (100%)	70,377 (96.8)
Race (%) (66,613 (91.65%))	
WHITE	35,216 (52.9%)
BLACK OR AFRICAN AMERICAN	27,081 (40.7%)
HISPANIC	3,101 (4.7%)
OTHER	1,215 (1.8%)
Albumin g/dl (mean (sd)) (69,982 (96.28%))	3.96 (0.46)
Alkaline phosphatase U/L (mean (sd)) (71,530 (98.41%))	86.11 (38.01)
ALT U/L (mean (sd)) (70,422 (96.89%))	61.64 (58.14)
APRI (mean (sd)) (100%)	0.62 (0.55)
AST U/L (mean (sd)) (100%)	49.94 (32.64)
Bilirubin mg/dl (mean (sd)) (72,115 (99.22%))	0.69 (0.41)
Body-mass-index (mean (sd)) (67,317 (92.62%))	27.17 (5.32)
Creatinine mg/dl (mean (sd)) (71,427 (98.27%))	1.08 (0.87)
INR (mean (sd)) (41,423 (56.99%))	1.06 (0.30)
Platelet count 1000/uL (mean (sd)) (100%)	233.02 (74.88)
Sodium mmol/L (mean (sd)) (72,271 (99.43%))	138.85 (3.24)

### Model performance

The average concordance over 30 splits for the CS Cox model without variable selection is 0.746, and the average concordance for the longitudinal Cox model without variable selection is 0.764. The average, standard deviation and 95% confidence intervals of concordance and AuROC for 1 year, 3 years, 5 years for the two models are provided in **Table 2**. The longitudinal Cox model outperformed the CS Cox model under all these measures, which are significantly different under paired t-tests.

**Table 2:** Performance of Cox models.

Cox Model*	Concordance (N = 21,805)	AuROC 1 year (N = 18,849)	AuROC 3 years (N = 14,602)	AuROC 5 years (N = 11,310)
**Cross-** **sectional Model**	0.746 (0.003) CI: 0.745–0.747	0.801 (0.008) CI: 0.799–0.804	0.784 (0.005) CI: 0.783–0.786	0.774 (0.003) CI: 0.772–0.776
**Longitudinal Model**	0.764 (0.003) CI: 0.763–0.766	0.820 (0.007) CI: 0.818–0.823	0.803 (0.005) CI: 0.802–0.805	0.794 (0.003) CI: 0.793–0.796
**P-value**	2*e-29	1*e-22	6*e-25	1*e-28

* mean (standard deviation), 95% confidence interval

The average concordance over 30 splits for the CS boosting model is 0.758, while the average concordance for the longitudinal boosting model is 0.774. The results for concordance and AuROC for 1 year, 3 years, 5 years for the two models are provided in **Table 3**.

**Table 3:** Performance of boosting models.

Boosting Model*	Concordance (N = 21,805)	AuROC 1 year (N = 18,849)	AuROC 3 years (N = 14,602)	AuROC 5 years (N = 11,310)
**Cross-** **sectional Model**	0.758 (0.003) CI: 0.757–0.759	0.811 (0.008) CI: 0.809–0.814	0.797 (0.005) CI: 0.795–0.799	0.787 (0.005) CI: 0.785–0.789
**Longitudinal Model**	0.774 (0.003) CI: 0.773–0.775	0.830 (0.007) CI: 0.827–0.832	0.814 (0.005) CI: 0.812–0.815	0.805 (0.004) CI: 0.804–0.807
**P-value**	8*e-28	4*e-24	3*e-23	1*e-26

* mean (standard deviation), 95% confidence interval

The longitudinal boosting model had better performance than the CS boosting model, and all four measures were significantly different based on results of paired t-tests. Further, with both longitudinal predictors and CS predictors, the boosting method performed better than the corresponding Cox model. This is expected because boosting methods can capture the nonlinearity in the data.

We also investigated properties such as sensitivity, specificity, NPV and PPV for 1 year, 3 years and 5 years predictions with the best cut-off under one representative training and testing split of the data. From a clinical standpoint, the utility of these predction models are often most useful for optimizing the NPV and thus cut-offs the maximized NPV were selected.The split is selected as the one whose longitudinal boosting model has the closest concordance to the average of 30 splits. The misclassification table for all four models under this split is provided in **Table 4**.

**Table 4:** Misclassification Table.

Time	Test Sample Size	Event Proportion	Model	AuROC	Best cut-off	Specificity	Sensitivity	PPV	NPV
**1 year**	18896	0.036	CS Cox	0.807	0.041	0.79	0.71	0.11	0.99
			CS Boosting	0.817	0.037	0.77	0.73	0.11	0.99
			LGT Cox	0.828	0.037	0.75	0.76	0.10	0.99
			LGT Boosting	0.838	0.035	0.76	0.77	0.11	0.99
**3 years**	14605	0.112	CS Cox	0.784	0.095	0.73	0.72	0.25	0.95
			CS Boosting	0.799	0.091	0.76	0.71	0.27	0.95
			LGT Cox	0.804	0.095	0.75	0.74	0.27	0.96
			LGT Boosting	0.815	0.090	0.76	0.73	0.28	0.96
**5 years**	11334	0.206	CS Cox	0.775	0.151	0.74	0.70	0.41	0.90
			CS Boosting	0.790	0.138	0.75	0.70	0.42	0.91
			LGT Cox	0.794	0.151	0.75	0.71	0.42	0.91
			LGT Boosting	0.805	0.128	0.73	0.74	0.41	0.92

(CS) cross-sectional; (LGT) longitudinal; (PPV) positive predictive value; (NPV) negative predictive value.

#### Variable selection for Cox models

We performed variable selection for the Cox models using elastic net penalty on 30 splits of data. For the CS Cox model, on average 14 predictors were selected out of the original 24 predictors. The average concordance of a non-penalized model on the selected variables was 0.745, which is very close to the performance of the original model. For the longitudinal Cox model, on average 29 predictors are selected out of the original 114 predictors. The average concordance of the non-penalized model after selection is 0.765, which is very close to that of the original model. The variable selection frequency in the 30 splits is provided in S1 Table and S2 Table. Variables that are selected in all 30 splits appear more relevant in the model, while those never or rarely selected seem to be marginal. Overall, the results suggest that with much fewer variables, we can still achieve comparable performance in the Cox models, which can be helpful for the adoption of the methods in clinical practice.

#### Importance and effect of predictors in boosting models

**Fig 3:**
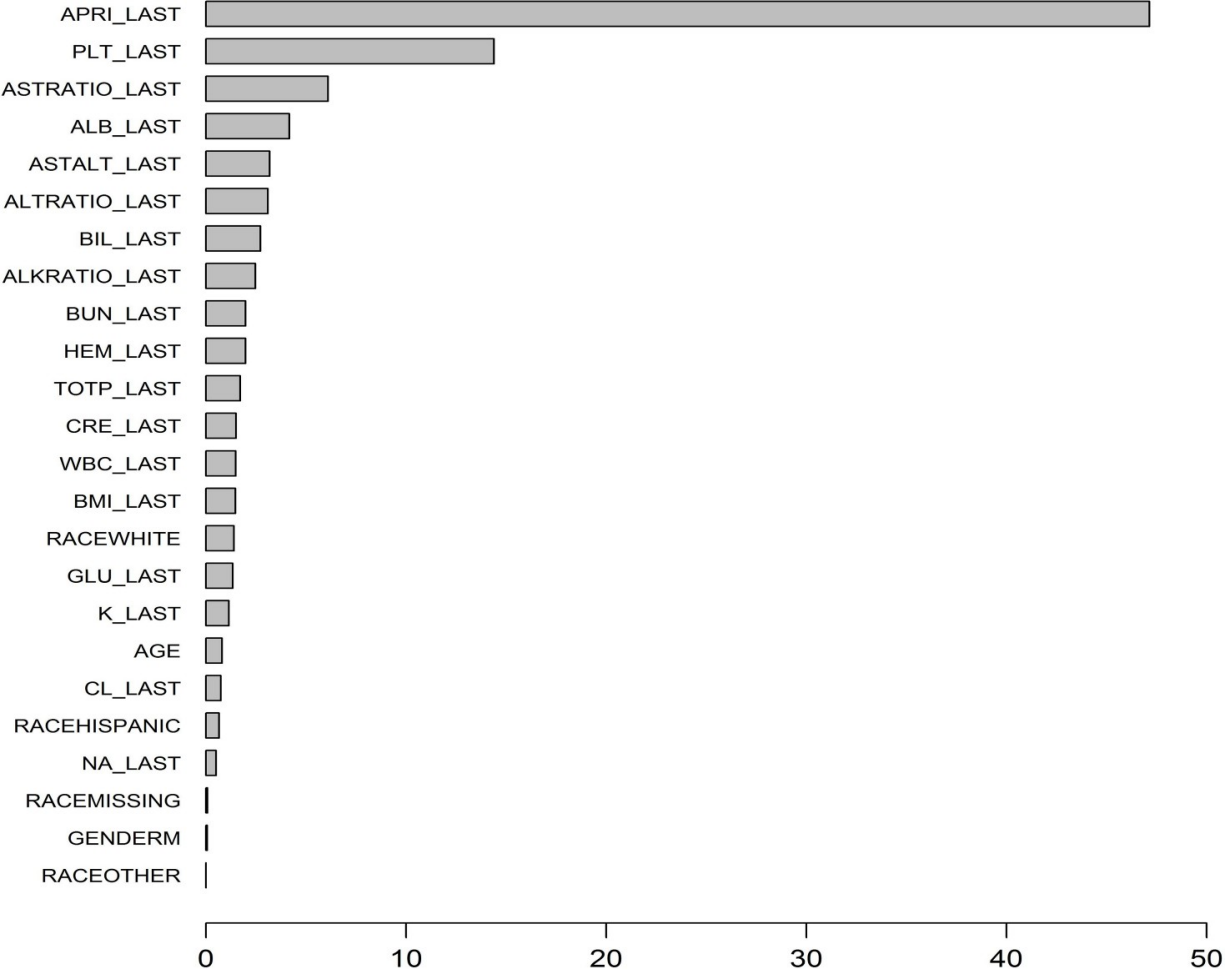
Variable importance plot for cross-sectional boosting model.

**Fig 4:**
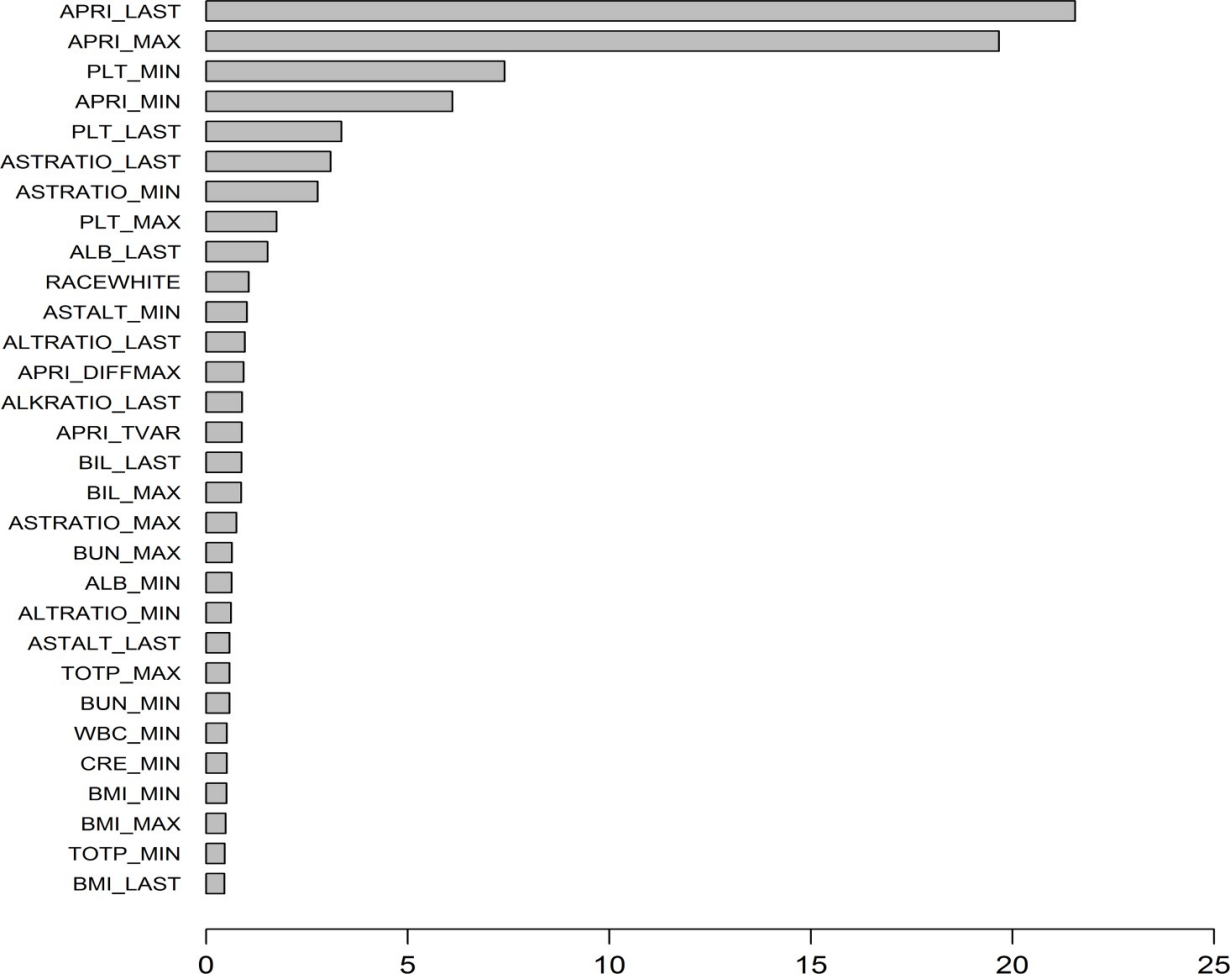
Variable importance plot for longitudinal boosting model.

The variable importance plot for the CS boosting model is provided in **Fig 3**. The five most important variables for distinguishing patients’ cirrhosis progression are as follows: APRI, PLT, AST ratio, ALB, AST/ALT. The variable importance plot for the longitudinal boosting model is provided in **Fig 4**. The five most important predictors are as follows: last APRI, maximum of APRI, minimum of PLT, minimum of APRI, last PLT.

**Fig 5:**
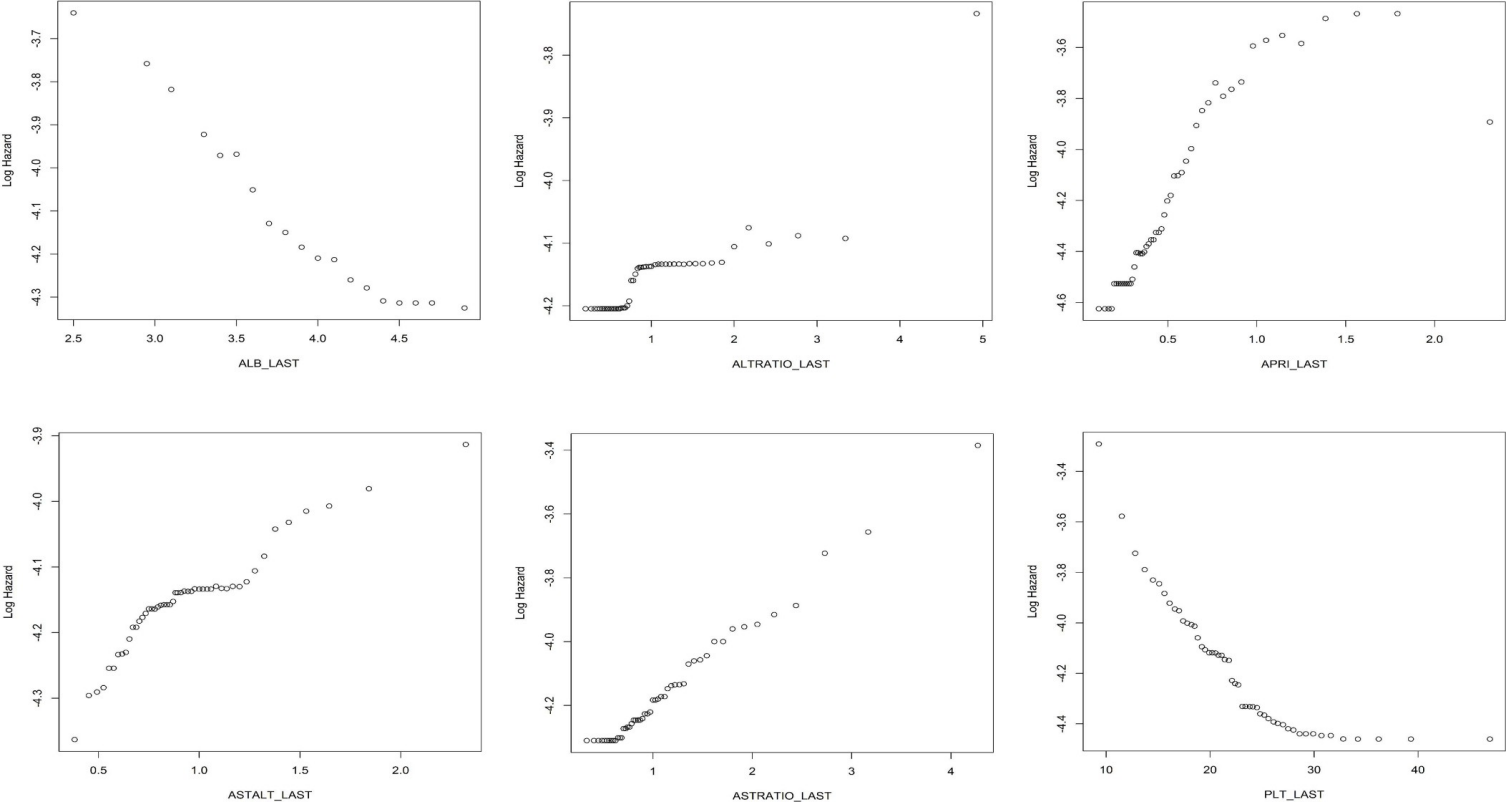
Partial dependence plots for the cross-sectional boosting model.

**Fig 6:**
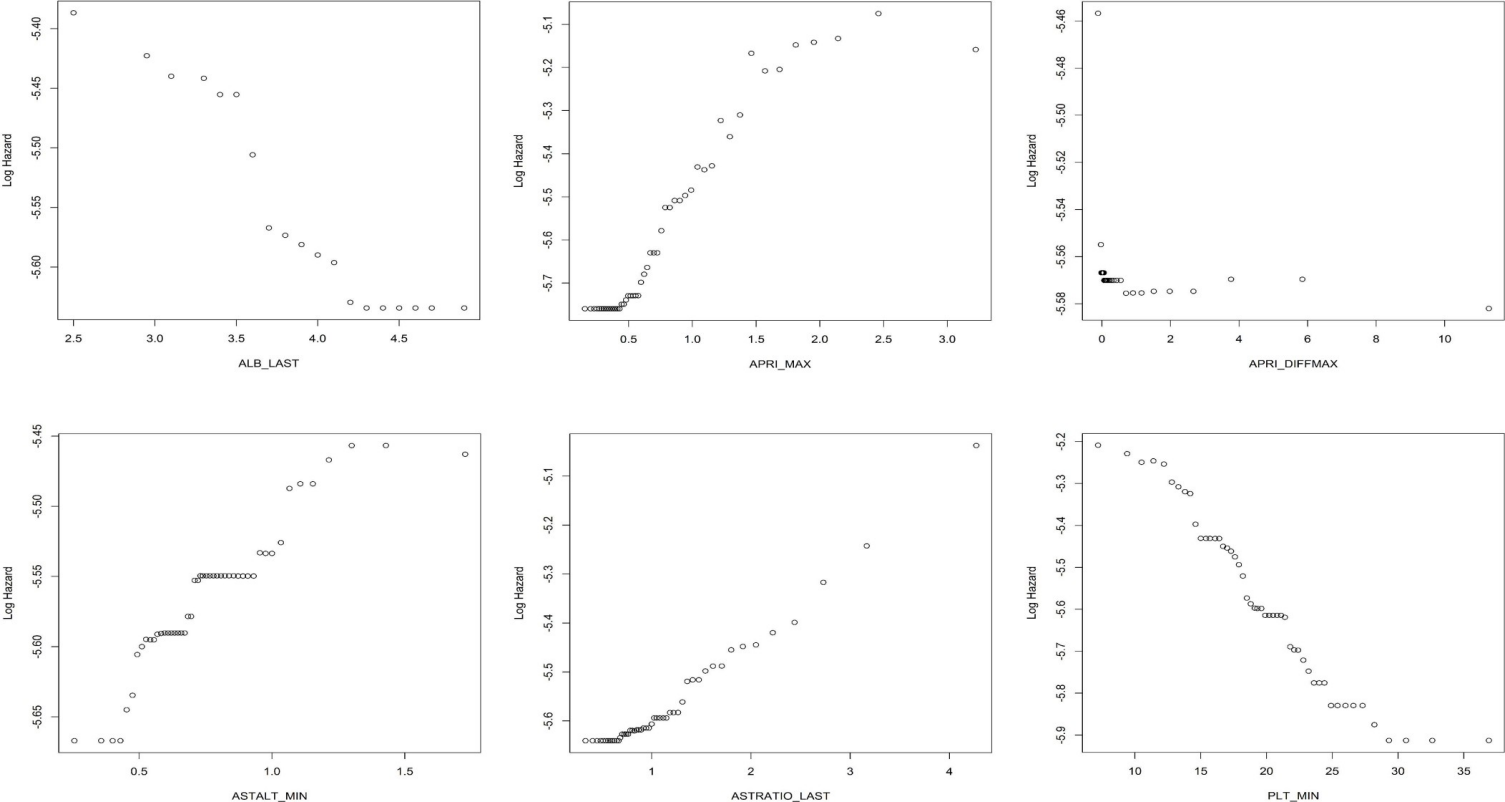
Partial dependence plots for the longitudinal boosting model.

The effects of individual predictors to the log hazard in the boosting models can be illustrated in partial dependence plots. The plots for selected important variables are provided in multiple panels of **Fig 5** for the CS boosting model, and in **Fig 6** for the longitudinal boosting model. We present the partial dependence plots of the top six important predictors (in terms of variable importance) for each model. If variables from a single lab appears more than once in the top predictors (e.g. PLT_MIN, PLT_LAST), we choose the one that has a clearer pattern in the partial dependence plot. We also include APRI_DIFFMAX, which has relatively large importance and also illustrates the effect of using slopes. It is important to realize that clinically, many laboratory values do not follow a linear relationship with the development of fibrosis or cirrhosis, (for example, albumin may be normal when you develop cirrhosis or vice-versa), and thus the individual results are often less informative than trends of longitudinal data.

## Discussion

HCV remains a significant public health problem despite the availability of highly effective antiviral treatment. Many health systems, such as the VHA, have been highly successful in treating patients with CHC in the direct-acting antiviral (DAA) treatment era. However, in the population as a whole, challenges persist that prevent disease eradication due to deficiencies in screening, access to care, and new or repeat infections related to the ongoing opioid epidemic and the high cost of anti-viral treatment.[22] Reflecting these barriers, it is estimated that only a minority of patients, estimated to be as low as 3%, on Medicaid have completed antiviral treatment.[23] Rates of acute HCV infection have also increased 2 fold in the past decade and is expected to increase in younger adults given the increase use of substance abuse in the population.[24] As such, risk prediction models that can assess for likelihood of disease progression remain relevant to many untreated and newly diagnosed patients. More importantly, application of these types of statistical modeling designs, particularly ML based algorithms, can potentially be applied to other chronic diseases with variable rates of progression.

In this study we were able to design, validate and compare several ML prediction models using a large cohort of patients. We demonstrate that it is feasible to create highly accurate models for risk of progression to cirrhosis at varying time intervals of interest. We show that while models based on longitudinal information are more accurate in their risk prediction, models built using only CS data are also accurate and thus represent a pragmatic approach for risk stratification in clinical settings. Through this analysis we also demonstrate the strengths of ML algorithms for non-linear, dynamic disease states. In addition, we applied ML methods for time-to-event analyses which to date has not been done extensively in large clinical cohorts.

This study has several important limitations. The primary limitation stems from the fact that VA cohorts differ from the larger overall cohort of patients with CHC. Our cohort consisted primarily of middle aged males and thus our results are most applicable to that subset of patients with CHC. However, 2700 women were included in our cohort allowing modeling of female sex as a predictor and potential inclusion in our models. Our analyses were intentionally restricted to patients who had never received antiviral treatment for HCV because we recognize that after antiviral therapy, the disease trajectory changes dramatically and will require different prediction models which we plan to address in a separate study. In addition, antiviral treatment may affect laboratory values and thus impact their predictive capability. Patients who have not completed antiviral therapy are also most representative of the natural disease course in CHC. In the DAA era, large percentages of patients have been successfully treated and thus assessment of risk of progression post SVR is an area of great interest to us which we are pursuing. This paper, however, addresses large cohort of patients prior to eradication of HCV in whom risk assessment is important as there remains real world barriers to universal treatment and an understanding of risk would help determine those who are most need of urgent treatment. Regarding potential statistical limitations, Cox models only account for linear effects of predictors, therefore we also constructed boosted-survival-tree based proportional hazards models which can incorporate non-linear effects of variables as well as the time-to-event nature of the data. We did not include other comorbid illness such as diabetes or alcohol use because prior models did not rank these comorbid illnesses high on the variable importance list. In addition, we wanted to use commonly available data that were easily extracted from the medical record. Finally, it is also worth highlighting the issue of missing data in this longitudinal study. Overall the percentage of missing data was quite small (2.4% on average for CS predictors and maximum/minimum longitudinal variables for each lab and 10.9% on average for maximum/minimum of slope and total variation variables for each lab), and we were able to address this using median imputation methods that were shown to have good performance characteristics.

In conclusion, ML based risk prediction models are highly accurate in assessing risk of fibrosis progression in a large cohort of veterans with CHC. Although algorithms based on longitudinal data had the most robust performance characteristics, CS models were similarly high performing and thus represent a less complex method to implement into clinical practice. ML methods applied to time-to-event analyses are an attractive statistical approach when assessing for risk of outcomes longitudinally in dynamic disease states.

## Supporting information

S1 Table Variable selection frequency for cross-sectional Cox model. (DOCX)

S2 Table Variable selection frequency for longitudinal Cox model. (DOCX)

## Abbreviations

ML: Machine Learning
IBD: Inflammatory bowel disease
CHC: chronic hepatitis C
VHA: Veterans Health Administration
APRI: Aspartate aminotransferase-to-platelet ratio index
CS: Cross-sectional
AuROC: Area under the Receiver Operating Characteristic Curve
HCV: Hepatitis C virus
HALT-C: Hepatitis C Antiviral Therapy to Prevent Cirrhosis
VistA: Veterans Information Systems and Technology Architecture
CDW: Corporate Data Warehouse
RNA: Ribonucleic acid
SVR: Sustained virologic response
ULN: Upper limit of normal
ALT: Alanine aminotransferase
ALK: Alkaline phosphatase
ALB: Albumin
BIL: Total bilirubin
CRE: Creatinine
BUN: Blood urea nitrogen
GLU: Glucose
HEM: Hemoglobin
PLT: Platelet count
WBC: White blood cell count
NA: Sodium
K: Potassium
CL: Chloride
TOTP: Total protein
BMI: Body mass index
DAA: Direct-acting antiviral
LGT: Longitudinal
NPV: Negative Predictive Value
PPV: Positive Predictive Value
