# Supplementary material for: Machine learning models to predict disease progression among veterans with hepatitis C virus
Source: PLoS One. 2019 Jan 4;14(1):e0208141. doi: 10.1371/journal.pone.0208141 (PMC6319806; doi:10.1371/journal.pone.0208141)

**Supporting Information**

**Supporting Table 2. Variable selection frequency for longitudinal Cox model**


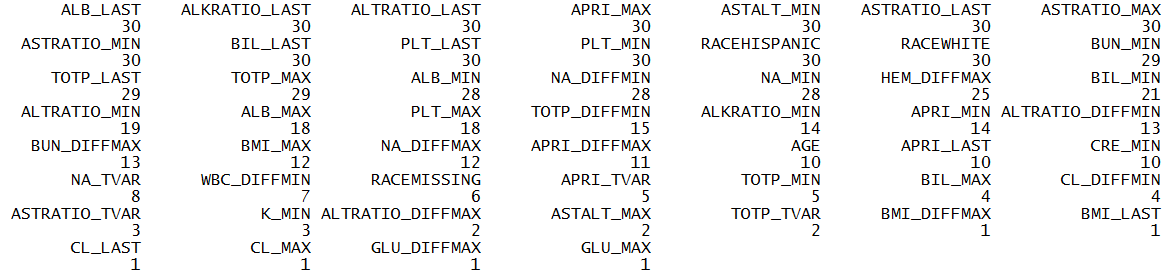

Supplement: S1 Table — (DOCX) [file pone.0208141.s001.docx]
